# Associations Between Maternal and Cord Blood Leptin Levels and Anthropometry of Mothers and Infants

**DOI:** 10.3390/nu18183105

**Published:** 2026-09-21

**Authors:** Hans Demmelmair, Johanna Bruder, Sultan Nilay Can, Jeannie Horak, Martin Klingenspor, Oraporn Dumrongwongsiri, Berthold Koletzko

**Affiliations:** 1 Dr. Von Hauner Children’s Hospital, LMU University Hospital, Ludwig Maximilians Universität Munich, Lindwurmstrasse 4, 80337 Munich, Germany; hans.demmelmair@med.uni-muenchen.de (H.D.); jeannie.horak@med.uni-muenchen.de (J.H.); 2 German Center for Child and Adolescent Health (DZKJ), 80337 Munich, Germany; 3 Chair for Molecular Nutritional Medicine, TUM School of Life Sciences, Technical University of Munich, 85354 Freising, Germany; 4 EKFZ-Else Kröner Fresenius Center for Nutritional Medicine, Technical University of Munich, 85354 Freising, Germany; 5 ZIEL Institute for Food & Health, Technical University of Munich, 85354 Freising, Germany; 6 Division of Nutrition, Department of Pediatrics, Faculty of Medicine Ramathibodi Hospital, Mahidol University, Bangkok 10400, Thailand; oraporn.roj@mahidol.ac.th; 7 Department Pediatrics, Chulalongkorn University, Bangkok 10330, Thailand

**Keywords:** pregnancy, leptin, birth weight, pre-pregnancy BMI

## Abstract

Background/Objectives: As birth weight is related to later obesity risk, its associations with maternal pre-pregnancy body mass index (BMI) and leptin values in serum during pregnancy are of interest for obesity prevention. This study aimed to describe the association of leptin levels in maternal circulation and cord blood as well as pre-pregnancy BMI (pBMI) with infant anthropometry at birth and until the age of four months. Methods: Data on maternal pBMI, at delivery and four months after birth, and on infant weight and length at delivery and at age four months were collected in an observational study in Bangkok, Thailand. In a subgroup of 38 mother–infant dyads, leptin was measured in maternal blood serum at delivery and infant age 4 months and in cord and infant serum at 4 months. Associations were tested with correlation and multiple linear regression analyses in this explorative study. Results: The maternal serum leptin was 32.4 ± 17.8 ng/mL at delivery and 11.1 ± 8.9 ng/mL at four months (*p* < 0.001 for difference) and correlated (r = 0.45). Cord serum leptin was significantly higher in female than male infants (17.5 ± 9.8 ng/mL vs. 7.7 ± 4.9 ng/mL, *p* < 0.05) and not related to infant leptin at age four months. There was no significant association between maternal and cord serum leptin levels. Birth weight was strongly associated with pBMI (standardized β = 0.77, *p* < 0.001) and inversely related to maternal delivery leptin levels (standardized β = −0.44, *p* = 0.008), while the association with cord serum leptin was not significant. Conclusions: Our findings indicate that pBMI is strongly related to birth weight, while higher maternal leptin concentrations were independently associated with lower birth weight after accounting for pBMI. Cord serum leptin did not significantly correlate with maternal leptin levels and only tended to be positively associated with birth weight.

## 1. Introduction

In recent decades, obesity prevalence has markedly increased among adults and children in affluent and in low- and middle-income countries [1,2]. While multiple environmental and individual risk factors contribute to obesity, energy intake and satiety regulation are major risk determinants [3]. Leptin plays a key role in obesity-related physiological processes [4]. Leptin is an anorexigenic peptide hormone acting on hypothalamic centers, also regulating energy expenditure [5]. The major source of leptin is adipose tissue, and leptin levels are positively associated with the amount of body fat [4].

The prenatal period is of specific importance for later trajectories of body weight and body composition [6]. Maternal pre-pregnancy BMI (pBMI) and gestational weight gain are positively associated with birth weight, postnatal weight gain and later obesity risk [7]. A meta-analysis including 66 studies confirmed that high birth weight increases later overweight risk [8]. During pregnancy, maternal serum leptin levels increase, which is ascribed more to placental leptin secretion than to increasing maternal fat stores during pregnancy [9].

While cord blood leptin is accepted as an indicator of fetal fat stores, the physiological effects of fetal leptin have not yet been fully elucidated [9]. Nevertheless, significant roles of leptin during pregnancy have been established, including indicating that there may be more effects on embryo implantation, placental angiogenesis, neurodevelopmental programming of metabolism, growth, immune modulation and nutrient transfer across the placenta [10,11,12].

The impact of maternal leptin levels during pregnancy and cord blood leptin on offspring growth and body weight development, in addition to the effects of birth weight, has been explored in several cohort studies. In the Project Viva cohort, Boeke et al. found that maternal leptin negatively associated with the offspring body mass index (BMI) z-score at 3 and 7 years of life [13]. In German mothers, infant weight gain until the age of one year was positively associated with cord blood leptin, while associations with maternal leptin were inverse [14]. In a Greek cohort, each increase in cord blood leptin of one standard deviation was associated with a more than 200 g lower weight at age four years in the total study population, but higher cord leptin was associated with higher weight in the subgroup of infants born with low weight for gestational age and those who showed rapid early postnatal weight gain [15]. Other studies reported positive associations of cord leptin with later obesity; however, associations may differ depending on offspring age [16]. Brunner et al. found negative associations with infant weight and BMI at birth and partially at later time points until the age of two years, mainly with maternal leptin but also with cord blood leptin [17].

These observations indicate different effects of maternal and cord blood leptin levels, which are not closely correlated with each other. In this context, contradictory findings have been reported [18]. A two-compartment model describing isolated pools of maternal and fetal leptin was postulated by Laml et al. based on the observation of the low transplacental transfer of leptin in vitro [19,20,21]. Quantification of placental leptin transfer in vivo has not been possible so far; thus, a close correlation of maternal and fetal leptin can be neither postulated nor excluded.

There is a considerable number of studies that have investigated leptin levels in the perinatal period, but the number of studies considering the association between maternal and cord leptin with infant anthropometry together with pBMI is limited. We aimed to test how maternal and infant leptin levels four months after delivery, a time when all infants are still breastfed, relate to levels at delivery or cord blood. Furthermore, studies on maternal weight, birth weight and postnatal weight gain including leptin levels have mainly been performed in Western populations. We aimed to study the association of pBMI, birth weight and infant early growth considering both maternal and cord blood leptin levels in an urban population in Thailand.

## 2. Materials and Methods

Serum samples and data from mothers and their infants from the prospective observational Thai Milk Study were collected in the metropolitan region of Bangkok, Thailand [22]. Ethical approval for this study was obtained from the Ethic Committee, Faculty of Medicine Ramathibodi Hospital, Mahidol University (ID 03-60-31) and the Ethical Committee, Ludwig Maximillian Universität, Munich (project No. 18-015) [22]. All participants gave written consent to participate after detailed explanation of the procedures.

Anthropometric measurements of mothers and infants were performed according to well-established standard operation procedures as described earlier [22]. Maternal serum samples were collected during pregnancy (upon study enrolment), prior to delivery (upon hospital admission for delivery) and four months (117–136 days) after delivery without consideration of prandial state. Infant serum was obtained at birth from cord blood (mixture of venous and arterial blood) and at the age of 4 months (117–136 days). The studied subset of 38 mother–infant pairs was selected from the total of 117 participants enrolled in the original study based on the availability of serum samples from each of the time points, with the exception of serum samples collected during pregnancy.

### 2.1. Measurement of Maternal and Child Serum Leptin Levels

Blood samples were centrifuged after sampling to separate serum, which was frozen and stored at –80 °C, until shipment to Munich, Germany, and further processing. Maternal and infant serum leptin samples were measured using a commercially available ELISA kit (Human Leptin DuoSet ELISA, DY398-05, Lot#P311723, R&D Systems, Minneapolis, MN, USA) according to the manufacturer’s instructions. For the color reagent, a ready-to-use tetramethylbenzidine solution (00-4201-56, Invitrogen, Thermo Fisher Scientific, Waltham, MA, USA) was purchased. Frozen serum samples were thawed on ice. All samples were mixed vigorously and centrifuged for 5 min at 4 °C and 200× *g* before further processing. Pipetting steps except plate washing were performed using a Microlab VANTAGE 1.3 pipetting robot from Hamilton (Bonaduz, Switzerland). Samples were measured at 50× dilution, but, for some samples, the dilution had to be adjusted to 10× or 200× to obtain measurements within the range of the standard curve.

### 2.2. Statistical Analyses

Results are presented as means and corresponding standard deviations. Kolmogorov–Smirnov tests were applied to test for normal distribution, and, if a variable was found not normally distributed, values were log-transformed. T-tests were applied for group comparisons. Pearson correlation coefficients and corresponding 95% confidence intervals (Fischer’s r-to-z transformation) were calculated to test associations. Multiple linear regression was applied to consider various influencing factors simultaneously. Variance inflation factors were used to test for collinearity, and only models with factors below 3 were included in the reported results. Statistical significance was assumed at *p*-values ≤ 0.05. Statistical analyses were performed using SPSS Version 31 (IBM Corp, Armonk, New York, NY, USA).

As the number of included subjects was based on the available samples, a power calculation was not performed; therefore, findings must be considered explorative and hypothesis-generating rather than for hypothesis testing. Applying Fisher’s z-transformation it can be estimated that true correlations of 0.44 or higher can be detected with a power of 80% at a two-tailed significance level of α = 0.05 from the 38 sample pairs available for our study.

## 3. Results

Thirty-eight mother–infant dyads were included in this leptin study. The characteristics, anthropometric measures and leptin levels of the dyads are presented in Table 1. The studied subgroup did not differ significantly in any of the parameters shown in Table 1 from those of the full study population (Supplemental Table S1) and included six mothers diagnosed with gestational diabetes. Infant birth weight was significantly higher in the group with than in the group without gestational diabetes (3507 ± 322 g vs. 3077 ± 329 g), but all other parameters studied were not significantly different.

Cord blood leptin was more than twofold higher in female than in male infants, but sex was not closely associated with anthropometric measures. Due to the sex differences in cord leptin, correlations between the various leptin concentrations and anthropometric measures are presented stratified for sex (Table 2).

**Figure 1:**
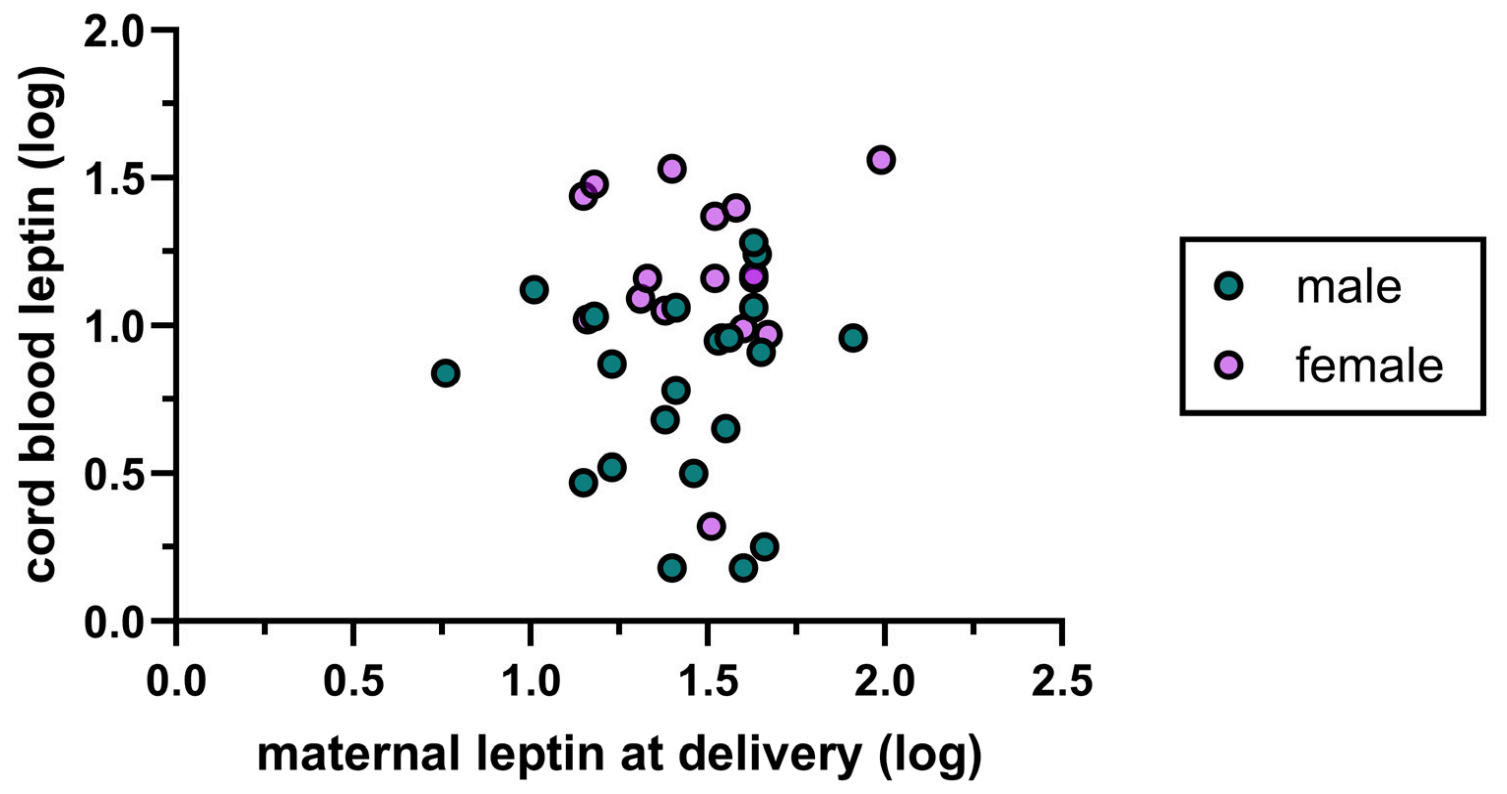
Maternal and cord blood leptin levels (log-transformed) at delivery were not correlated.

A positive correlation of pBMI with maternal leptin at delivery was also found for females and males, whereas the positive correlation with cord blood leptin was not robust when stratified by sex (Table 2). Birth weight and ponderal index were significantly positively associated with cord blood leptin in males, while this relationship was not significant in females. There was no significant correlation between maternal leptin at delivery and cord blood leptin (Figure 1, Table 2).

We tested the combined influence of pBMI, sex, gestational age, pregnancy weight gain and leptin levels on birth weight with a multiple linear regression approach in the total study population (Supplemental Table S2). Birth weight was chosen as it provided closer associations than the ponderal index. In none of the models, neither sex, gestational age, nor gestational weight gain was a significant factor. Therefore, the final model included only pBMI and leptin levels (Table 3).

**Figure 2:**
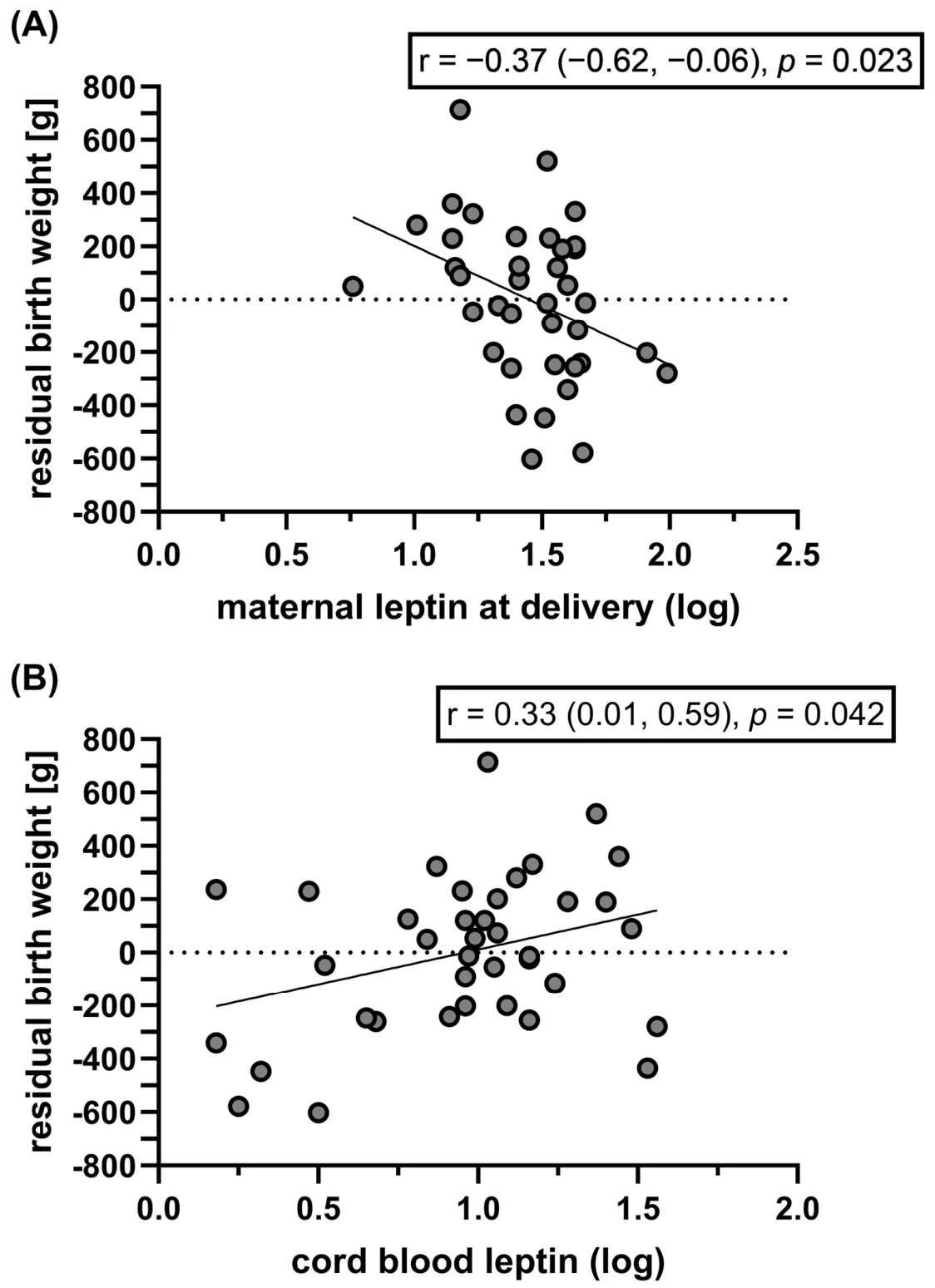
Scatter plots showing residuals of birth weight regressed on pBMI versus log-transformed maternal leptin (**A**) and cord blood leptin (**B**).

Maternal pBMI was positively associated with birth weight, while maternal leptin levels at delivery showed a negative association with birth weight. As maternal adiposity and fetal growth may be different from healthy pregnancies in mothers with gestational diabetes, the regression analysis was also performed excluding the six mothers with gestational diabetes (Table 4). Excluding mothers with gestational diabetes basically did not change the findings. The relationships of maternal and cord leptin with birth weight are visualized in Figure 2, showing the residuals of the regression of birth weight on pBMI versus the leptin levels.

To investigate the effects of leptin levels on postnatal weight development, Pearson correlation coefficients were calculated between log-transformed maternal and cord blood leptin and postnatal weight and BMI z-scores at the ages of two and four months. No significant correlations were found. Birth weight positively correlated with weight and BMI z-score at both two and four months (r-values above 0.41). Regression models considering birth weight as a confounder showed no associations of leptin levels with postnatal weight or BMI z-score at the age of two, but weight at 4 months was significantly associated with cord serum leptin, although not with the BMI z-score (Supplemental Table S3). Infant leptin at four months was significantly lower than in cord serum (*p* < 0.001, paired *t*-test) and did not correlate with cord blood leptin.

Maternal pBMI closely correlated with maternal BMI at four months after delivery (r = 0.93 [0.87, 0.96]). At four months, maternal leptin values were significantly lower than at delivery (*p* < 0.001, paired *t*-test), and the values were significantly correlated (r = 0.45, 95–CI 0.15–0.67).

Except for two mothers with a pBMI of almost 30 or higher, all other pBMI values ranged from 15 to 25 kg/m^2^. As a sensitivity analysis, the regression model was run after the exclusion of these two mothers with high pBMI values (Supplemental Table S4). The very similar results indicate that findings did not depend on these two mothers.

## 4. Discussion

In this Thai study cohort, cord blood leptin levels correlated with infant birth weight. Multiple regression analysis identified maternal pBMI and maternal leptin, but not cord blood leptin, as related to birth weight. Infant weight and BMI z-scores at the ages of two and four months were influenced by birth weight, but only a weak direct association with cord leptin was detected.

In our cohort of 38 mother–infant dyads, the positive correlations between maternal pBMI and infant birth weight and pBMI and cord blood leptin agree with those of previous studies [23,24], but they were not robust after stratification by sex. The close relationship of pBMI and maternal leptin in our study agrees with observations during pregnancy and in non-pregnant humans, as well as with adipose tissue being the major source of circulating leptin [14,18,21,25,26,27,28,29]. Nevertheless, leptin concentrations increase in pregnant women during pregnancy [30]. Placental leptin production, increased adipose tissue mass and more intense leptin secretion from adipose tissue due to hormonal changes contribute to the higher levels [31]. Perfused placenta experiments did not provide indications that placental leptin secretion correlates with maternal BMI at delivery [31], but in vitro studies show that most leptin synthesized in the placenta is secreted into the maternal circulation [31,32]. The models do not enable the determination of the relative importance of placenta as a source of total leptin in maternal or fetal circulation; thus, a positive association between pBMI and maternal leptin seems plausible.

In our cohort, infant sex was a major determinant of cord blood leptin. This agrees with the significantly higher leptin levels in female infants observed in other studies [33,34] and with pBMI being positively associated with cord blood leptin [18]. Leptin production in fetal adipose tissues has been reported from gestational week 20 onwards [35]. Estimation of fetal weight and fetal adiposity via 2D ultrasonography at gestational weeks 30 and 36, together with measurement of cord blood leptin, indicated that leptin levels are not necessarily related to fetal weight but rather to fetal adiposity [33]. As higher pre-pregnancy weight is associated with higher relative and absolute neonatal fat [36], this suggests a positive correlation between pBMI and cord blood leptin.

Although. in our study, maternal and fetal leptin were related to pBMI, there was no significant correlation between the two leptin pools. This finding is in line with several other studies that also have reported no significant association [14,17,18,19,26,27,30]. However, some studies identified moderately strong and significant positive correlations [28,29,37,38]. The transfer of leptin across the placenta was low in model experiments [20,21], which explains the gradient of leptin concentrations between maternal and cord blood leptin. Thus, it is understandable that the correlations between maternal and fetal pools are not strong. We and others have observed associations of infant sex with cord leptin levels but not with maternal levels, which again reflects low placental leptin transfer and contributes to weakening the correlation between maternal and cord blood levels. The placenta produces leptin and exports it to the fetal and maternal sides, but no correlation was found between the export to the two pools [31]. Some studies have identified significant differences between venous and arterial cord blood, apparently influenced by placental leptin production [39]. In our study, we used samples containing both venous and arterial cord blood, which may have introduced additional variation in cord blood leptin concentration. The available evidence overall suggests no or only weak correlations between maternal and cord leptin levels and a moderate influence of pBMI on both levels. Therefore, the two-pool model for maternal and cord blood leptin, as suggested by Laml, seems justified [19].

We found that maternal leptin negatively associated and cord blood leptin no longer significantly positively associated with birth weight when considered jointly with pBMI. A Polish study including 168 mothers with a wide variation in pBMI found that pBMI, but not gestational weight gain, positively related to birth weight, whereas maternal leptin tended towards a negative association with birth weight (n.s.) [40]. Papastefanou et al. found a significant negative correlation between first trimester leptin and birth weight z-scores in healthy and pre-eclamptic pregnancies [41]. In a study with four serial measurements of maternal leptin distributed across all trimesters, Hinkle et al. found a good correlation between the leptin levels at the different time points, indicating that the observed associations between maternal leptin and birth weight do not depend on the time point in pregnancy when leptin is measured, although the mechanisms behind the association might change with the course of pregnancy [42]. Leptin levels from all collection time points (10–14 weeks, 15–26, 23–31, 33–39) tended to be negatively associated with birth weight, but statistical significance was only found for the last time point and was limited to the subgroup of women with a pBMI < 30 kg/m^2^ [42]. In agreement with our study, Brunner et al. found that maternal leptin negatively associated with birth weight after adjustment for maternal pBMI [17]. It was beyond the scope of our study to explore the mechanisms, but our observation agrees with a study of 740 mothers reporting a significant negative association of birth weight standard deviation scores with maternal leptin that determined BMI at enrolment as a confounder [43]. The authors of this large study speculated that higher leptin levels might be associated with a lowering of the transplacental nutrient transfer, which could limit fetal growth [43].

A meta-analysis including 44 studies identified a moderate significant correlation between birth weight and cord blood leptin (average r = 0.46), with no differences between male and female infants or between Caucasian and Asian populations [24]. The degree of this association is similar to the r-value of 0.48 in our study population. Although not significant, cord blood leptin also tended to be positively associated with birth weight in our regression model including pBMI and maternal leptin. A significant positive association of log-transformed cord leptin with birth weight adjusted for pBMI has already been observed in the large Rhea cohort and in other studies [18,19,25,27,29,44]. Of interest, our data indicate that the positive association between maternal pBMI and birth weight is accompanied by an independent association of higher maternal leptin concentrations with lower birth weight when accounting for maternal pre-pregnancy BMI. It is tempting to speculate that placental leptin production, which mostly enters the maternal circulation, contributes to this effect, although it did not increase with maternal BMI at delivery [31]. Although maternal leptin levels at four months after delivery correlated with values at delivery, they decreased on average by more than 60% after delivery. This observation agrees with previous findings and the assumption of a contribution of placental leptin to circulating maternal leptin levels during pregnancy but does not necessarily indicate a correlation between placental and other maternal tissue production [30,45,46].

The postnatal anthropometric data in our study show the expected association between birth weight and weight at later time points, but there was no association with maternal leptin and only a weak association of cord leptin with infant weight at four months. This finding is comparable to other studies, which observed some, but inconsistent, associations between maternal or cord blood leptin and growth or anthropometry parameters during the first months of postnatal life [17,47]. In mouse pups, intraperitoneal leptin injections from postnatal day 17 onwards increased metabolic rates and led to decreased food intake only at day 28, which might explain the more marked effects of prenatal leptin on offspring adiposity in humans only being observed at later ages, from 2 to 5 years [48,49].

A major limitation of our study is the small sample size, which limited exploration of potential confounders including sex, made a longer-term follow up unfeasible, precluded the reliable detection of modest associations, and increased the risk of random observations. Due to the explorative nature of our study, correction for multiple testing was not performed, requiring critical evaluation of the identified statistical significances. Although no significant differences between included and not included study participants could be identified, the inclusion of 38 (full study population of 117) subjects based on sample availability might have introduced selection bias. Additional limitations are that mixed arterial and venous cord blood was used, residual confounding could not be excluded, and the definition of the statistical models and variable transformation may have influenced the results. Although sensitivity analyses showed stability of the findings, the inclusion of women with gestational diabetes or extreme values may have influenced our results. Also, we did not have placenta weight or neonatal body composition data, which could have been helpful in interpreting our observations given that placental and fetal adipose tissue are relevant sources of leptin production during pregnancy. A strength of our study is the established standard operation procedures for data collection, with the high precision of the data. We could extend the observations made so far in Western population to mothers in Thailand, who have different ethnic and dietary backgrounds.

## 5. Conclusions

Our explorative study identified a positive association between maternal pBMI and birth weight as well as an inverse association between maternal leptin and birth weight, in agreement with previous studies. Also in agreement with other findings, cord blood leptin was not closely related to maternal leptin levels but could be an indicator of fetal adipose tissue and may have further downstream effects. The observed complex involvement of leptin in early development emphasizes the need for larger prospective studies, adequately powered to test hypotheses, relating leptin during pregnancy with birth weight and offspring growth, and enabling comprehensive adjustment for maternal metabolic and obstetric factors.

## Figures and Tables

**Table 1:** Characteristics, anthropometric data and serum leptin levels of mother–infant dyads (mean ± SD); values for male and female infants were compared by *t*-test. * *p* ≤ 0.05; ^a^ pre-pregnancy BMI.

	Total	Male (n = 21)	Female (n = 17)
pre-pregnancy weight (kg)	54.2 ± 9.6	51.5 ± 7.4	57.5 ± 11.2
pBMI ^a^ (kg/m^2^)	21.93 ± 3.8	21.09 ± 2.84	22.98 ± 4.61
BMI mother at 4 months (kg/m^2^)	23.20 ± 4.33	22.39 ± 3.20	24.21 ± 5.35
gestational weight gain (kg)	15.0 ± 4.3	15.0 ± 3.9	15.0 ± 4.8
gestational age (weeks)	38.6 ± 1.2	38.5 ± 1.3	38.8 ± 1.0
birth weight (g)	3145 ± 360	3106 ± 315	3194 ± 415
birth length (cm)	49.9 ± 1.6	49.6 ± 1.4	50.2 ± 1.7
infant weight at 2 months (g)	5325 ± 613	5394 ± 562	5241 ± 678
infant weight at 4 months (g)	6798 ± 819	6972 ± 778	6583 ± 840
infant ponderal index at birth (kg/m^3^)	25.38 ± 2.75	25.46 ± 2.71	25.29 ± 2.88
infant ponderal index at 2 months (kg/m^3^)	26.91 ± 2.45	27.28 ± 2.27	26.45 ± 2.65
infant ponderal index at 4 months (kg/m^3^)	26.09 ± 2.33	26.34 ± 2.37	25.77 ± 2.31
maternal leptin at delivery (ng/mL)	32.4 ± 17.8	31.2 ± 16.8	33.8 ± 19.4
maternal leptin at 4 months (ng/mL)	11.1 ± 8.9	9.7 ± 5.4	12.7 ± 11.9
cord blood leptin (ng/mL)	12.1 ± 8.9	7.7 ± 4.9 *	17.5 ± 9.8 *
infant leptin at age 4 months (ng/mL)	5.1 ± 2.9	5.0 ± 2.4	5.2 ± 3.4

**Table 2:** Statistically significant Pearson correlations (95% CI) between measured leptin values (log-transformed) and anthropometric parameters of mothers and infants, stratified for infant sex (* *p* ≤ 0.05, ** *p* ≤ 0.01). ^a^ Pre-pregnancy BMI, ^b^ infant ponderal index at birth, ^c^ maternal leptin at delivery.

Male (n = 21)	Birth Weight	Ponderal-Index ^b^	Cord Leptin	Maternal Leptin ^c^
pBMI ^a^	ns	0.64 (0.29–0.84) **	ns	0.69 (0.37–0.86) **
birth weight		0.65 (0.31–0.85) **	0.51 (0.11–0.77) *	ns
ponderal index ^b^			0.47 (0.05–0.75) *	ns
cord blood leptin				ns
**Female (n = 17)**	**Birth Weight**	**Ponderal-Index ^b^**	**Cord Leptin**	**Maternal Leptin ^c^**
pBMI ^a^	0.78 (0.48–0.92) **	ns	ns	0.63 (0.22–0.85) **
birth weight		0.60 (0.16–0.84) *	ns	ns
ponderal index ^b^			ns	ns
cord blood leptin				ns
**Total (n = 38)**	**Birth Weight**	**Ponderal-Index ^b^**	**Cord Leptin**	**Maternal Leptin ^c^**
pBMI ^a^	0.58 (0.32–0.76) **	0.43 (0.13–0.66) **	0.36 (0.04–0.61) **	0.63 (0.38–0.79) **
birth weight		0.61 (0.36–0.78) **	0.48 (0.18–0.69) *	ns
ponderal index ^b^			0.32 (0.00–0.58) *	ns
cord leptin				ns

**Table 3:** Multiple linear regression model relating pBMI to birth weight (outcome) considering maternal and cord blood leptin (log-transformed) as further potentially influencing factors (n = 38).

	Beta (95% CI)	Standardized Beta	*p*-Value	R^2^
pBMI	73 (41, 105)	0.77	<0.001	0.53
cord blood leptin	226 (−32, 484)	0.23	ns	
maternal leptin	−655 (−1126, −184)	−0.44	0.008	

**Table 4:** Multiple linear regression model relating pBMI to birth weight (outcome) considering maternal and cord blood leptin (log transformed) as additional potentially influencing factors excluding mothers with gestational diabetes (n = 32); ^a^ pre-pregnancy BMI.

	Beta (95% CI)	Standardized Beta	*p*-Value	R^2^
pBMI ^a^	72 (39, 105)	0.81	<0.001	0.53
cord blood leptin	179 (−65, 424)	0.21	ns	
maternal leptin	−562 (−1028, −96)	−0.43	0.020	

## Data Availability

Data can be made available for legitimate research purposes upon request to the authors.

## Supplementary Materials

The following supporting information can be downloaded at: https://www.mdpi.com/article/10.3390/nu18183105/s1, Table S1. Characteristics and anthropometric data of the mother-infant dyads (mean ± SD) included into this study compared to the dyads not included, groups were compared by *t*-test, all *p*-values > 0.06. Table S2. Multiple linear regression model relating pBMI, maternal and cord blood leptin (log transformed), sex, gestational age and gestational weight gain to the outcome birth weight (n = 38). Table S3. Multiple linear regression model relating post natal weight and BMI-zscore at the ages of two and four months (outcomes) to maternal and cord blood leptin (log transformed) including birth weight as a confounder (n = 38). Table S4. Multiple linear regression model relating pBMI to birth weight (outcome) considering maternal and cord blood leptin (log transformed) as further potentially influencing factors including only mothers with a prepregnancy BMI below 26 kg/m^2^ (n = 36).

## Abbreviations

The following abbreviation is used in this manuscript:

pBMI	Pre-pregnancy body mass index

## References

[1] Zhang X., Liu J., Ni Y., Yi C., Fang Y., Ning Q., Shen B., Zhang K., Liu Y., Yang L. (2024). Global Prevalence of Overweight and Obesity in Children and Adolescents: A Systematic Review and Meta-Analysis. JAMA Pediatr..

[2] NCD-Risk-factor-Collaboration (2024). Worldwide trends in underweight and obesity from 1990 to 2022: A pooled analysis of 3663 population-representative studies with 222 million children, adolescents, and adults. Lancet.

[3] Dhurandhar N.V., Petersen K.S., Webster C. (2021). Key Causes and Contributors of Obesity: A Perspective. Nurs. Clin. N. Am..

[4] Mantzoros C.S., Moschos S.J. (1998). Leptin: In search of role(s) in human physiology and pathophysiology. Clin. Endocrinol..

[5] Skoracka K., Hryhorowicz S., Schulz P., Zawada A., Ratajczak-Pawłowska A.E., Rychter A.M., Słomski R., Dobrowolska A., Krela-Kaźmierczak I. (2025). The role of leptin and ghrelin in the regulation of appetite in obesity. Peptides.

[6] Larque E., Labayen I., Flodmark C.E., Lissau I., Czernin S., Moreno L.A., Pietrobelli A., Widhalm K. (2019). From conception to infancy—early risk factors for childhood obesity. Nat. Rev. Endocrinol..

[7] Zheng M., Hesketh K.D., Vuillermin P., Dodd J., Wen L.M., Baur L.A., Taylor R., Byrne R., Mihrshahi S., Burgner D. (2023). Understanding the pathways between prenatal and postnatal factors and overweight outcomes in early childhood: A pooled analysis of seven cohorts. Int. J. Obes..

[8] Schellong K., Schulz S., Harder T., Plagemann A. (2012). Birth weight and long-term overweight risk: Systematic review and a meta-analysis including 643,902 persons from 66 studies and 26 countries globally. PLoS ONE.

[9] Hauguel-de Mouzon S., Lepercq J., Catalano P. (2006). The known and unknown of leptin in pregnancy. Am. J. Obstet. Gynecol..

[10] Valencia-Ortega J., Castillo-Santos A., Molerés-Orduña M., Solis-Paredes J.M., Saucedo R., Estrada-Gutierrez G., Camacho-Arroyo I. (2024). Influence of Maternal Adipokines on Anthropometry, Adiposity, and Neurodevelopmental Outcomes of the Offspring. Int. J. Mol. Sci..

[11] Pérez-Pérez A., Toro A., Vilariño-García T., Maymó J., Guadix P., Dueñas J.L., Fernández-Sánchez M., Varone C., Sánchez-Margalet V. (2018). Leptin action in normal and pathological pregnancies. J. Cell. Mol. Med..

[12] Bouret S.G., Lucas A.M.M., Ziegler E.E. (2010). Leptin, nutrition, and the programming of hypothalamic feeding circuits. Nestle Nutr Workshop Ser Pediatr Program.

[13] Boeke C.E., Mantzoros C.S., Hughes M.D., Rifas-Shiman S.L., Villamor E., Zera C.A., Gillman M.W. (2013). Differential associations of leptin with adiposity across early childhood. Obesity.

[14] Telschow A., Ferrari N., Deibert C., Flöck A., Merz W.M., Gembruch U., Ehrhardt C., Dötsch J., Graf C. (2019). High Maternal and Low Cord Blood Leptin Are Associated with BMI-SDS Gain in the First Year of Life. Obes. Facts.

[15] Karakosta P., Roumeliotaki T., Chalkiadaki G., Sarri K., Vassilaki M., Venihaki M., Malliaraki N., Kampa M., Castanas E., Kogevinas M. (2016). Cord blood leptin levels in relation to child growth trajectories. Metabolism.

[16] Blais K., Doyon M., Arguin M., Bouchard L., Perron P., Hivert M.F. (2022). Associations between Cord Blood Leptin Levels and Childhood Adiposity Differ by Sex and Age at Adiposity Assessment. Life.

[17] Brunner S., Schmid D., Huttinger K., Much D., Bruderl M., Sedlmeier E.M., Kratzsch J., Amann-Gassner U., Bader B.L., Hauner H. (2014). Effect of reducing the n-6/n-3 fatty acid ratio on the maternal and fetal leptin axis in relation to infant body composition. Obesity.

[18] Tsai P.J., Davis J., Bryant-Greenwood G. (2015). Systemic and placental leptin and its receptors in pregnancies associated with obesity. Reprod. Sci..

[19] Laml T., Hartmann B.W., Ruecklinger E., Preyer O., Soeregi G., Wagenbichler P. (2001). Maternal serum leptin concentrations do not correlate with cord blood leptin concentrations in normal pregnancy. J. Soc. Gynecol. Investig..

[20] Wyrwoll C.S., Mark P.J., Waddell B.J. (2005). Directional secretion and transport of leptin and expression of leptin receptor isoforms in human placental BeWo cells. Mol. Cell. Endocrinol..

[21] Briffa J.F., McAinch A.J., Romano T., Wlodek M.E., Hryciw D.H. (2015). Leptin in pregnancy and development: A contributor to adulthood disease?. Am. J. Physiol. Endocrinol. Metab..

[22] Dumrongwongsiri O., Winichagoon P., Chongviriyaphan N., Suthutvoravut U., Grote V., Koletzko B. (2021). Effect of Maternal Nutritional Status and Mode of Delivery on Zinc and Iron Stores at Birth. Nutrients.

[23] Voerman E., Santos S., Inskip H., Amiano P., Barros H., Charles M.A., Chatzi L., Chrousos G.P., Corpeleijn E., Crozier S. (2019). Association of Gestational Weight Gain With Adverse Maternal and Infant Outcomes. JAMA.

[24] Karakosta P., Chatzi L., Plana E., Margioris A., Castanas E., Kogevinas M. (2011). Leptin levels in cord blood and anthropometric measures at birth: A systematic review and meta-analysis. Paediatr. Perinat. Epidemiol..

[25] Geary M., Pringle P.J., Persaud M., Wilshin J., Hindmarsh P.C., Rodeck C.H., Brook C.G. (1999). Leptin concentrations in maternal serum and cord blood: Relationship to maternal anthropometry and fetal growth. Br. J. Obstet. Gynaecol..

[26] Helland I.B., Reseland J.E., Saugstad O.D., Drevon C.A. (1998). Leptin levels in pregnant women and newborn infants: Gender differences and reduction during the neonatal period. Pediatrics.

[27] Lazo-de-la-Vega-Monroy M.L., González-Domínguez M.I., Zaina S., Sabanero M., Daza-Benítez L., Malacara J.M., Barbosa-Sabanero G. (2017). Leptin and its Receptors in Human Placenta of Small, Adequate, and Large for Gestational Age Newborns. Horm. Metab. Res..

[28] Manderson J.G., Patterson C.C., Hadden D.R., Traub A.I., Leslie H., McCance D.R. (2003). Leptin concentrations in maternal serum and cord blood in diabetic and nondiabetic pregnancy. Am. J. Obstet. Gynecol..

[29] Stefaniak M., Dmoch-Gajzlerska E., Mazurkiewicz B., Gajzlerska-Majewska W. (2019). Maternal serum and cord blood leptin concentrations at delivery. PLoS ONE.

[30] Schubring C., Englaro P., Siebler T., Blum W.F., Demirakca T., Kratzsch J., Kiess W. (1998). Longitudinal analysis of maternal serum leptin levels during pregnancy, at birth and up to six weeks after birth: Relation to body mass index, skinfolds, sex steroids and umbilical cord blood leptin levels. Horm. Res..

[31] Linnemann K., Malek A., Sager R., Blum W.F., Schneider H., Fusch C. (2000). Leptin production and release in the dually in vitro perfused human placenta. J. Clin. Endocrinol. Metab..

[32] Hoggard N., Crabtree J., Allstaff S., Abramovich D.R., Haggarty P. (2001). Leptin secretion to both the maternal and fetal circulation in the ex vivo perfused human term placenta. Placenta.

[33] Tamai J., Ikenoue S., Akita K., Hasegawa K., Otani T., Fukutake M., Kasuga Y., Tanaka M. (2025). Association of Cord Blood Metabolic Biomarkers (Leptin, Adiponectin, IGF-1) with Fetal Adiposity Across Gestation. Int. J. Mol. Sci..

[34] Tome M.A., Lage M., Camiña J.P., Garcia-Mayor R.V., Dieguez C., Casanueva F.F. (1997). Sex-based differences in serum leptin concentrations from umbilical cord blood at delivery. Eur. J. Endocrinol..

[35] Lepercq J., Challier J.C., Guerre-Millo M., Cauzac M., Vidal H., Hauguel-de Mouzon S. (2001). Prenatal leptin production: Evidence that fetal adipose tissue produces leptin. J. Clin. Endocrinol. Metab..

[36] Catalano P.M. (2007). Management of obesity in pregnancy. Obstet. Gynecol..

[37] Logan C.A., Bornemann R., Koenig W., Reister F., Walter V., Fantuzzi G., Weyermann M., Brenner H., Genuneit J., Rothenbacher D. (2017). Gestational Weight Gain and Fetal-Maternal Adiponectin, Leptin, and CRP: Results of two birth cohorts studies. Sci. Rep..

[38] Ökdemir D., Hatipoğlu N., Kurtoğlu S., Siraz Ü.G., Akar H.H., Muhtaroğlu S., Kütük M.S. (2018). The Role of Irisin, Insulin and Leptin in Maternal and Fetal Interaction. J. Clin. Res. Pediatr. Endocrinol..

[39] Yura S., Sagawa N., Mise H., Mori T., Masuzaki H., Ogawa Y., Nakao K. (1998). A positive umbilical venous-arterial difference of leptin level and its rapid decline after birth. Am. J. Obstet. Gynecol..

[40] Mazurek D., Bronkowska M. (2020). Maternal Anthropometric Factors and Circulating Adipokines as Predictors of Birth Weight and Length. Int. J. Environ. Res. Public Health.

[41] Papastefanou I., Samolis S., Panagopoulos P., Tagia M., Bale C., Kouskoukis A., Galazios G. (2010). Correlation between maternal first trimester plasma leptin levels and birth weight among normotensive and preeclamptic women. J. Matern. Fetal Neonatal Med..

[42] Hinkle S.N., Rawal S., Liu D., Chen J., Tsai M.Y., Zhang C. (2019). Maternal adipokines longitudinally measured across pregnancy and their associations with neonatal size, length, and adiposity. Int. J. Obes..

[43] Serapio S., Ahlsson F., Larsson A., Kunovac Kallak T. (2019). Second Trimester Maternal Leptin Levels Are Associated with Body Mass Index and Gestational Weight Gain but not Birth Weight of the Infant. Horm. Res. Paediatr..

[44] Karakosta P., Georgiou V., Fthenou E., Papadopoulou E., Roumeliotaki T., Margioris A., Castanas E., Kampa M., Kogevinas M., Chatzi L. (2013). Maternal weight status, cord blood leptin and fetal growth: A prospective mother-child cohort study (Rhea study). Paediatr. Perinat. Epidemiol..

[45] Butte N.F., Hopkinson J.M., Nicolson M.A. (1997). Leptin in human reproduction: Serum leptin levels in pregnant and lactating women. J. Clin. Endocrinol. Metab..

[46] Andersson-Hall U., Svedin P., Svensson H., Lönn M., Mallard C., Holmäng A. (2020). Longitudinal changes in adipokines and free leptin index during and after pregnancy in women with obesity. Int. J. Obes..

[47] Schneider C.R., Catalano P.M., Biggio J.R., Gower B.A., Chandler-Laney P.C. (2018). Associations of neonatal adiponectin and leptin with growth and body composition in African American infants. Pediatr. Obes..

[48] Mistry A.M., Swick A., Romsos D.R. (1999). Leptin alters metabolic rates before acquisition of its anorectic effect in developing neonatal mice. Am. J. Physiol..

[49] Bagias C., Sukumar N., Weldeselassie Y., Oyebode O., Saravanan P. (2021). Cord Blood Adipocytokines and Body Composition in Early Childhood: A Systematic Review and Meta-Analysis. Int. J. Environ. Res. Public Health.

