# Supplementary material for: Associations Between Maternal and Cord Blood Leptin Levels and Anthropometry of Mothers and Infants
Source: Nutrients. 2026 Sep 21;18(18):3105. doi: 10.3390/nu18183105 (PMC13610258; doi:10.3390/nu18183105)
Supplement: Supplementary file 1 [file nutrients-18-03105-s001.zip › nutrients-4512110-supplementary.pdf]

Table S1. Characteristics and anthropometric data of the mother-infant dyads (mean ± SD) included into this study compared to the dyads not included, groups were compared by t-test, all p-values > 0.06.

	**Included (n = 38)**	**Not Included (n = 82)**
pre-pregnancy weight (kg)	54.2 ± 9.6	55.7 ± 10.7
pBMI (kg/m^2)	21.93 ± 3.8	22.02 ± 3.93
BMI mother 4 months (kg/m^2)	23.20 ± 4.33	23.41 ± 4.15
gestational weight gain (kg)	15.0 ± 4.3	13.3 ± 5.3
gestational age (weeks)	38.6 ± 1.2	38.4 ± 1.2
birth weight (g)	3145 ± 360	3070 ± 409
birth length (cm)	49.9 ± 1.6	49.7 ± 2.2
infant weight 2 month (g)	5325 ± 613	5211 ± 621
infant weight 4 month (g)	6798 ± 819	6501 ± 761
infant ponderal index birth (kg/m^3)	25.38 ± 2.75	25.09 ± 2.45
infant ponderal index 2 months (kg/m^3)	26.91 ± 2.45	27.33 ± 2.48
infant ponderal index 4 months (kg/m^3)	26.09 ± 2.33	25.96 ± 2.62
infant BMI 2 months (z-score)	-0.39 ± 1.00	-0.36 ± 0.99
infant BMI 4 months (z-score)	-0.27 ± 1.07	-0.49 ± 1.10

Table S2. Multiple linear regression model relating pBMI, maternal and cord blood leptin (log transformed), sex, gestational age and gestational weight gain to the outcome birth weight (n = 38). [a] 0 = female, 1 = male.

	**Beta (95% CI)**	**Standardized Beta**	**p-Value**	**R^2**
**pBMI**	78 (46, 110)	0.83	<0.001	0.59
**cord blood leptin**	280 (-24, 585)	0.29	ns	
**maternal leptin**	-780 (-1290, -368)	-0.52	0.004	
**gestational age**	23 (-57, 103)	0.07	ns	
**gestational weight gain**	14 (-8.3, 36)	0.17	ns	
**sex[a]**	135 (-63, 334)	0.19	ns	

Table S3. Multiple linear regression model relating post natal weight and BMI-zscore at the ages of two and four months (outcomes) to maternal and cord blood leptin (log transformed) including birth weight as a confounder (n = 38).

**weight at two months**				
	**beta (95% CI)**	**standardized beta**	**p-value**	**R^2**
**birth weight**	1.33 (0.79, 1.88)	0.72	<0.001	0.44
**cord blood leptin**	-453 (-950, 44)	-0.27	ns	
**maternal leptin**	168 (-540, 877)	0.48		

**weight at four months**				
	**beta (95% CI)**	**standardized beta**	**p-value**	**R^2**
**birth weight**	1.39 (0.52, 2.26)	0.54	0.003	0.28
**cord blood leptin**	-822 (-1611, -32)	-0.35	0.042	
**maternal leptin**	-337 (-1462, 787)	-0.09	ns	

**BMI-z-score at two months**				
	**beta (95% CI)**	**standardized beta**	**p-value**	**R^2**
**birth weight**	0.002 (0.001, 0.003)	0.57	0.001	0.29
**cord blood leptin**	-0.58 (-1.52, 0.36)	-0.20	ns	
**maternal leptin**	0.74 (-0.60, 2.07)	0.17	ns	

**BMI-z-score at four months**				
	**beta (95% CI)**	**standardized beta**	**p-value**	**R^2**
**birth weight**	0.002 (0.000, 0.003)	0.46	0.014	0.18
**cord blood leptin**	-0.69 (-1.77, 0.39)	-0.23	ns	
**maternal leptin**	0.27 (-1.27, 1.80)	0.06	ns	

Table S4. Multiple linear regression model relating pBMI to birth weight (outcome) considering maternal and cord blood leptin (log transformed) as further potentially influencing factors including only mothers with a prepregnancy BMI below 26 kg/m^2 (n = 36).

	**Beta (95% CI)**	**Standardized Beta**	**p-Value**	**R^2**
**pBMI**	78 (38, 118)	0.67	<0.001	0.44
**cord blood leptin**	232 (-22, 486)	0.26	ns	
**maternal leptin**	-607 (-1079, -134)	-0.43	0.013	
